# Radix Astragali Improves Dysregulated Triglyceride Metabolism and Attenuates Macrophage Infiltration in Adipose Tissue in High-Fat Diet-Induced Obese Male Rats through Activating mTORC1-PPAR**γ** Signaling Pathway

**DOI:** 10.1155/2014/189085

**Published:** 2014-04-08

**Authors:** Yang Long, Xiang-Xun Zhang, Tao Chen, Yun Gao, Hao-Ming Tian

**Affiliations:** ^1^Laboratory of Endocrinology and Metabolism, West China Hospital of Sichuan University, Chengdu, Sichuan 610041, China; ^2^Department of Endocrinology and Metabolism, West China Hospital of Sichuan University, Chengdu, Sichuan 610041, China

## Abstract

Increased levels of free fatty acids (FFAs) and hypertriglyceridemia are important risk factors for cardiovascular disease. The effective fraction isolated from radix astragali (RA) has been reported to alleviate hypertriglyceridemia. The mechanism of this triglyceride-lowering effect of RA is unclear. Here, we tested whether activation of the mTORC1-PPAR**γ** signaling pathway is related to the triglyceride-lowering effect of RA. High-fat diet-induced obese (DIO) rats were fed a high-fat diet (40% calories from fat) for 9-10 weeks, and 4 g/kg/d RA was administered by gavage. RA treatment resulted in decreased fasting triglyceride levels, FFA concentrations, and adipocyte size. RA treated rats showed improved triglyceride clearance and fatty acid handling after olive oil overload. RA administration could also decrease macrophage infiltration and expression of MCP-1 and TNF**α**, but it may also increase the expression of PPAR**γ** in epididymal adipose tissue from RA treated rats. Consistently, expressions of PPAR**γ** and phospho-p70S6K were increased in differentiated 3T3-L1 adipocytes treated with RA. Moreover, RA couldnot upregulate the expression of PPAR**γ** at the presence of rapamycin. In conclusion, the mTORC1-PPAR**γ** signaling pathway is a potential mechanism through which RA exerts beneficial effects on the disturbance of triglyceride metabolism and dysfunction of adipose tissue in DIO rats.

## 1. Introduction

Obesity is becoming one of the most serious public health problems worldwide. Substantial epidemiological evidence has proven that overweight and obesity raise the risk for chronic diseases, such as cardiovascular disease [1, 2], type 2 diabetes [3, 4], some cancers [5], and stroke [6]. Additionally, obesity is thought to be one of the top five global health risks for mortality, which accounts for 5% of deaths [7].

It has been well established that obesity is characterized by excessive expansion of adipose tissue, particularly visceral adipose tissue, and is accompanied by chronic low-grade inflammation in adipose tissue, which is triggered by nutrient excess. Inflammatory pathways are chronically activated in both adipocytes and the macrophages that reside in adipose tissue, resulting in substantial M1 macrophage infiltration and aberrant secretion of proinflammatory cytokines (such as TNF*α*, IL-6, and MCP-1) [8, 9]. Meanwhile, deranged triglyceride (TG) metabolism is partially a result of inflamed adipose tissue, resulting in hypertriglyceridemia and excessive FFA efflux from white adipose tissue [10, 11].

PPAR*γ*, a member of the nuclear hormone receptor superfamily, is a multifaceted molecule that is involved in initiating the terminal differentiation of adipocytes and in regulating the transcriptional expression of genes involved in lipid metabolism [12]. In addition to its established roles indicated above, PPAR*γ* is a potential anti-inflammatory factor. Thiazolidinediones, synthetic agonists of the ligand-activated transcription factor PPAR*γ*, suppress the expression of inflammatory cytokines in monocytes and macrophages [13]. One PPAR*γ* ligand, 15d-PGJ2, also inhibits gene expression of MCP-1 [14]. It has been well established that upregulated expression and activity of PPAR*γ* can exert a positive effect on dysregulated adipose tissue function associated with obesity. mTOR is an atypical serine/threonine protein kinase and is found in two functionally and structurally distinct multiprotein complexes, mTORC1 and mTORC2. It is believed that activation of mTORC1, composed of mTOR, raptor, mLST8, and PRAS40, promotes the expression of PPAR*γ* [15]. Constitutive activation of mTORC1 via loss of TSC2 increases PPAR*γ* expression [16]. Rapamycin, which inhibits the activity of the mTOR signaling pathway, has been reported to suppress the mRNA and protein levels of PPAR*γ* [17].

Radix astragali (RA), the dried roots of* Astragalus membranaceus* var.* mongholicus*, is a multifunctional traditional Chinese herb. Recently, it has been demonstrated that the effective fraction isolated from RA alleviates glucose intolerance, insulin resistance, and hypertriglyceridemia in db/db diabetic mice through its anti-inflammatory activity [18]. In addition, isoflavone, an effective fraction isolated from RA, is able to activate PPAR*γ in vitro* [19]. Therefore, we hypothesized that PPAR*γ* was a key molecule in the process of RA's improvements in dysregulated lipid metabolism and chronic inflammation in adipose tissue associated with high-fat diet-induced obesity. In this current study, our aim was to test our hypothesis and to identify whether there was interplay between mTORC1 and PPAR*γ* in the context of anti-inflammatory and improved triglyceride metabolism effects of RA. First, we explored the effect of RA on the derangement of triglyceride metabolism and inflamed adipose tissue in Sprague Dawley obese rats and observed whether RA regulated the expression of PPAR*γ*. Then, we evaluated whether the mTORC1 signaling pathway was involved in the upregulation of the expression of PPAR*γ* by RA.

## 2. Materials and Methods

### 2.1. Chemical Analysis by High-Performance Liquid Chromatography (HPLC)

Extract of RA was purchased from the DIAO Group (Chengdu, Sichuan, China). As indicated in the instruction provided by manufacturer, a 1 mL sample of extract of RA was prepared from 2 g RA. To identify the major constituents in the extract of RA, the extract sample was sent to Qingdao S&S Chemicals Analysing and Testing Co., Ltd., and HPLC was performed in an Ultimate 3000 system. The HPLC analysis results showed that polysaccharides and astragaloside are the two major constituents of the RA extraction. The extract of RA which we used to treat the high-fat diet-induced obese rats contained polysaccharides (518.29 mg/L), astragaloside (52.4 mg/L), calycosin (11.1 mg/L), and formononetin (1 mg/L).

### 2.2. Experimental Animals and Diet

Sprague Dawley male rats (DaShuo Laboratory Animal Co., Ltd., Chengdu, China) were maintained on a 12 h light/dark cycle at 24 ± 2°C. Rats had access to water and food ad libitum. Before the study, all rats were fed a standard chow (20% of calories from fat). For the diet-induced obesity experiment (HFD-C), 6-week-old rats were fed a high-fat diet (40% of calories from fat) for 20 weeks. For the RA experiment (HFD-RA), the rats were treated with 4 g/kg/d RA by oral gavage for 10 weeks following 10 weeks of a high-fat diet. Rats in the control group were fed standard chow for 20 weeks and treated with (NFD-RA) or without 4 g/kg/d RA (NFD-C). Weight gain during the experiments was monitored by weighing the rats every week.

After 20 weeks, the rats were sacrificed following a 5-hour fast and the intra-abdominal white adipose tissues (epididymal and perirenal fat pads) were weighed, then rapidly excised, and snap frozen in liquid nitrogen for subsequent experiments.

### 2.3. Metabolic Measurement

Blood samples were obtained from the four groups of rats after 20 weeks of the experiment. The blood was collected from the tail-tip of the rats after a 5-hour fast. Plasma glucose and triglyceride and FFA concentrations were measured using commercial assay kits. Fasting insulin concentrations were detected using a rat Insulin RIA kit (Linco Research Inc., St. Charles, USA). For the olive oil challenge, the rats were fasted for 5 hours and 10 *μ*L/g olive oil was administered by oral gavage, and then blood triglycerides and FFA concentrations were measured before and at 2, 4, 6. and 8 h after the olive oil gavage.

### 2.4. Cell Culture

3T3-L1 cells were cultured in high-glucose DMEM supplemented with 100 U of penicillin/mL, 100 *μ*g of streptomycin/mL, and 10% FBS in 5% CO_2_, 37°C incubator. The cells were allowed to grow for 2 more days after 100% confluency was reached; then, the cells were differentiated through the addition of differentiate medium (complete medium supplemented with 500 *μ*M isobutylmethylxanthine (Sigma-Aldrich Co., St. Louis, USA), 25 *μ*M dexamethasone (Sigma-Aldrich Co., St. Louis, USA), and 4 *μ*g/mL insulin (Eli Lilly and Company, Indianapolis, USA)) for 3 days and then complete medium with 4 *μ*g/mL insulin was added for an additional 3 days. The differentiated cells were maintained in complete medium for 6 additional days till the cells were fully differentiated.

### 2.5. Western Blotting

Cell lysates of epididymal adipose tissue and 3T3-L1 adipocytes were prepared in 300 *μ*L total volume of lysis buffer (Beyotime Institute of Biotechnology, Shanghai, China) on ice in 1.5 mL microtubes for 15 min and centrifuged for 5 min at 12,000 g at 4°C. The supernatant was collected and protein concentrations were measured using the Thermo Scientific Pierce BCA Protein Assay Kit (Pierce Biotechnology, Rockford, USA); then, the protein samples were stored at −80°C until further examination.

For the western blot, cell lysates were subjected to SDS-PAGE and immunoblotting was performed using specific antibodies against TNF*α* (Santa Cruz Biotechnology, Inc., Taxes, USA), MCP-1 (Santa Cruz Biotechnology, Inc., Taxes, USA), PPAR*γ* (Santa Cruz Biotechnology, Inc., Taxes, USA), p70S6K (Cell Signaling Technology, Inc., Boston, USA), phospho-p70S6K (Thr389) (Cell Signaling Technology, Inc., Boston, USA), and *β*-actin (ZSGB-Bio, Inc., Beijing, China).

### 2.6. Immunohistological Analysis

Paraffin-embedded epididymal adipose tissue was analyzed by H and E staining, and area measurement was performed in more than 20 representative images and 200 cells per rat. For estimating macrophage infiltration in the epididymal fat pad, immunohistological staining was performed and macrophages were detected using a F4/80 antibody (Santa Cruz Biotechnology, Inc., Taxes, USA) at a dilution of 1 : 50.

### 2.7. Quantitative Real-Time PCR

Total RNA was extracted from epididymal adipose tissue using Trizol reagent (Life Technologies Corporation, Carlsbad, California, USA) and RNA concentrations and purity were determined by measuring OD260, OD260/280 ratio, and OD260/230 ratio. RNA was reverse transcribed into cDNA using PrimeScript RT reagent kit with gDNA Eraser (Perfect Real Time) (TAKARA Biotechnology, (Dalian) Co., Ltd., Dalian, China). Quantitative real-time PCR analysis was performed using SsoFast EvaGreen Supermix (Bio-Rad Laboratories, Inc., California, USA). Primers used in the present study were shown as follows: MCP-1 (forward: 5′-TTTCCACAACCACCTCAAGC-3′, reverse: 5′-TGTTGAACCAGGATTCACAGAG-3′); PPAR*γ* (forward: 5′-CTGACCCAATGGTTGCTGATTAC-3′, reverse: 5′-CCTGTTGTAGAGTTGGGTTTTTTCA-3′); TNF*α* (forward: 5′-GTTGCCTCCCCCTTTTCTTT-3′, reverse: 5′-CCTGGTCACCAAATCAGCATT-3′); and GAPDH (forward: 5′-TCCCATTCTTCCACCTTTGATGCT-3′, reverse: 5′-ACCCTGTTGCTGTAGCCATATTCAT-3′).

### 2.8. Statistics

Data are expressed as mean ± SEM. Data were analyzed by 1-way ANOVA. Comparisons between time points were analyzed using repeated measures ANOVA. *P* values of less than 0.05 were considered statistically significant.

## 3. Results

### 3.1. RA Treatment Has No Effect on Body Weight and Intra-Abdominal White Adipose Tissue Mass but Decreases Adipocyte Size

**Figure 1:**
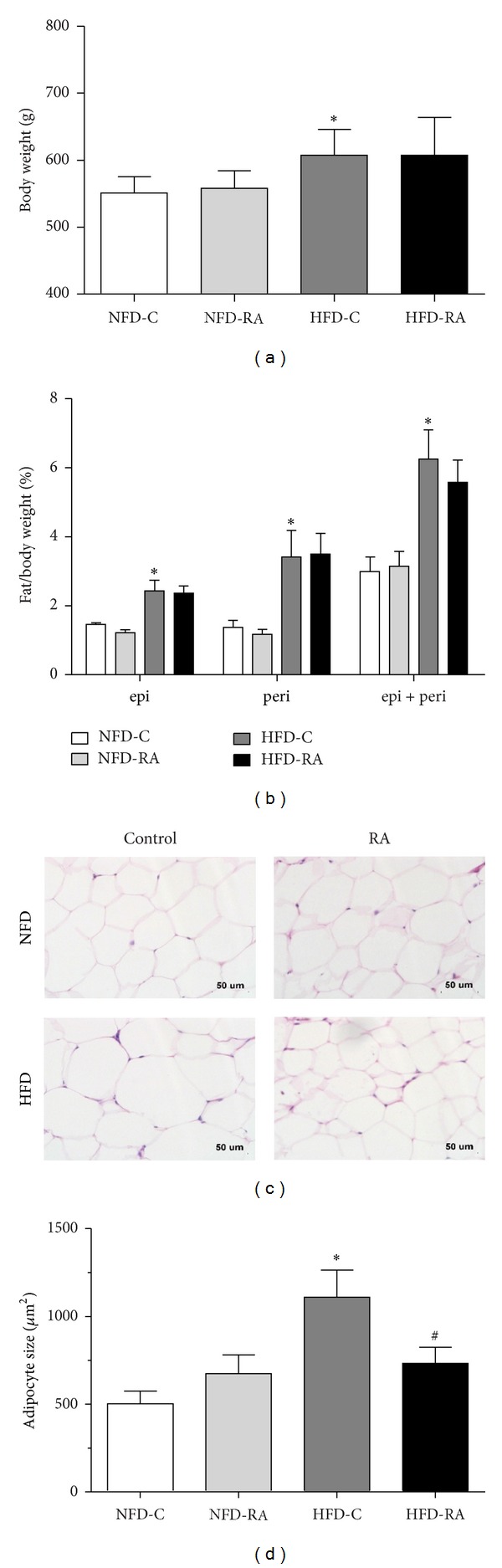
RA treatment has no effect on body weight and intra-abdominal white adipose tissue mass but decreases adipocyte size. (a) Body weights of rats fed standard chow without RA administration (NFD-C) and with RA administration (NFD-RA), high-fat diet without RA administration (HFD-C) and high-fat diet with RA administration (HFD-RA) (20 weeks, here and throughout, except as noted, *n* = 5–8/group) (b) epididymal (epi), perirenal (peri), and epi + peri fat pad weights (normalized to body weights here) in rats in the NFD-C, NFD-RA, HFD-C, and HFD-RA groups (20 weeks, *n* = 5–8/group). (c) Histology of epididymal adipose tissue from rats in the NFD-C, NFD-RA, HFD-C, and HFD-RA groups. Scale bar: 50 *μ*m. (d) Mean adipocyte size in rats in the NFD-C, NFD-RA, HFD-C, and HFD-RA groups (*n* = 5/group). *P < 0.05 (HFD-C group versus NFD-C group) and ^#^ *P* < 0.05 (HFD-RA group versus HFD-C group).

To assess the effect of RA on the development of obesity, we fed standard chow (20% calories derived from fat) or a high-fat diet (40% calories derived from fat) to SD rats and treated the rats with 4 g/kg/d RA by oral gavage following 10-week high-fat diet. As shown in Figure 1(a), SD rats, fed on the high-fat diet (HFD-C), displayed higher body weights than the control rats in the NFD-C and NFD-RA groups, which was 10% higher than the latter. The body weights of the rats in the HFD-RA group were comparable with those of the HFD-C group (Figure 1(a)). In addition to their similar body weights, there were no significant differences in the mass of the intra-abdominal white adipose tissue (normalized to body weights here) in either of the groups with or without RA gavage administration (Figure 1(b)). However, adipocyte size was decreased by 34% in the epididymal fat pad in the high-fat diet-fed rats treated with RA when compared with obese rats (Figures 1(c) and 1(d)).

### 3.2. RA Treatment Is Associated with Improvements in Lipid Metabolic Parameters of High-Fat Diet-Induced Obese Rats

**Figure 2:**
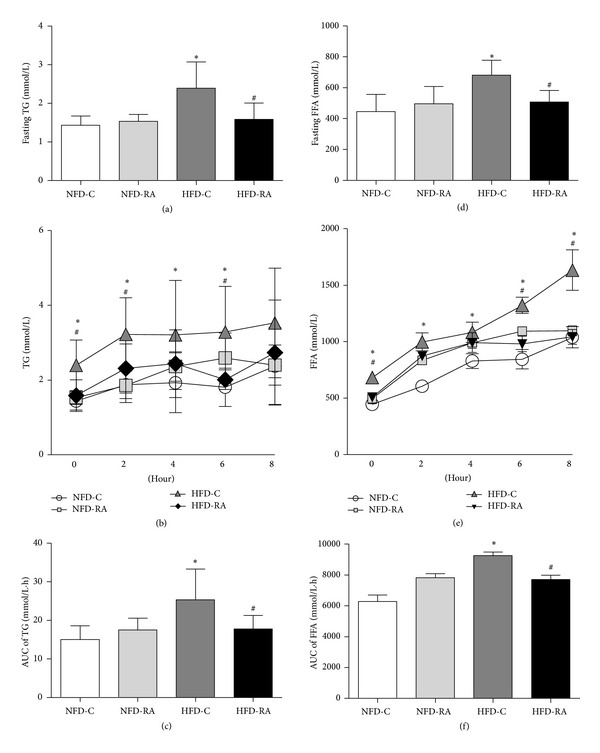
RA treatment decreases TG and FFA levels after a 5-hour fast and olive oil gavage. (a) TG concentrations in rats in the NFD-C, NFD-RA, HFD-C, and HFD-RA groups after a 5-hour fast (*n* = 5–8/group). (b) TG concentrations in rats in the NFD-C, NFD-RA, HFD-C, and HFD-RA groups after olive oil gavage (*n* = 5–8/group). (c) The AUC of TG levels during olive oil challenge is shown. (d) FFA concentrations in rats in the NFD-C, NFD-RA, HFD-C, and HFD-RA groups after a 5-hour fast (*n* = 5–8/group). (e) FFA concentrations in rats in the NFD-C, NFD-RA, HFD-C, and HFD-RA groups after olive oil gavage (*n* = 5–8/group). (f) AUC of FFA levels during olive oil challenge is shown. *P < 0.05 (HFD-C group versus NFD-C group) and ^#^ *P* < 0.05 (HFD-RA group versus HFD-C group).

To determine whether RA administration has an impact on the lipid metabolic parameters dysregulated in high-fat diet-induced obese rats, we detected fasting triglyceride and FFA levels. In contrast to the HFD-C group, the fasting serum triglyceride concentrations were reduced by 24% (Figure 2(a)) and the fasting plasma FFA concentrations significantly decreased by 22% in the rats in the HFD-RA group (Figure 2(d)).

To investigate the effect of RA on the dynamic handling of a lipid challenge, we performed a triglyceride challenge test using olive oil (10 *μ*L/g body weight) gavage after a 5-hour fast. As shown in Figure 2(b), the serum TG levels increased less at 2 hours after the lipid challenge and decreased more at 6 hours after the olive oil gavage in rats in the HFD-RA group than in the HFD-C group. Although the plasma FFA concentrations were comparable in rats treated with or without RA at 2 and 4 hours after lipid challenge, the FFA concentrations were significantly decreased in the rats treated with RA as compared with the obese rats without RA treatment at 6 and 8 hours after olive oil gavage (Figure 2(e)). The AUC values of FFA curve (Figure 2(c)) and triglyceride curve (Figure 2(f)) were both significantly decreased in RA therapy rats when compared with high-fat diet-induced obese rats.

### 3.3. RA Treatment Improves Chronic Inflammatory Activity in Intra-Abdominal White Adipose Tissue from Obese Rats

**Figure 3:**
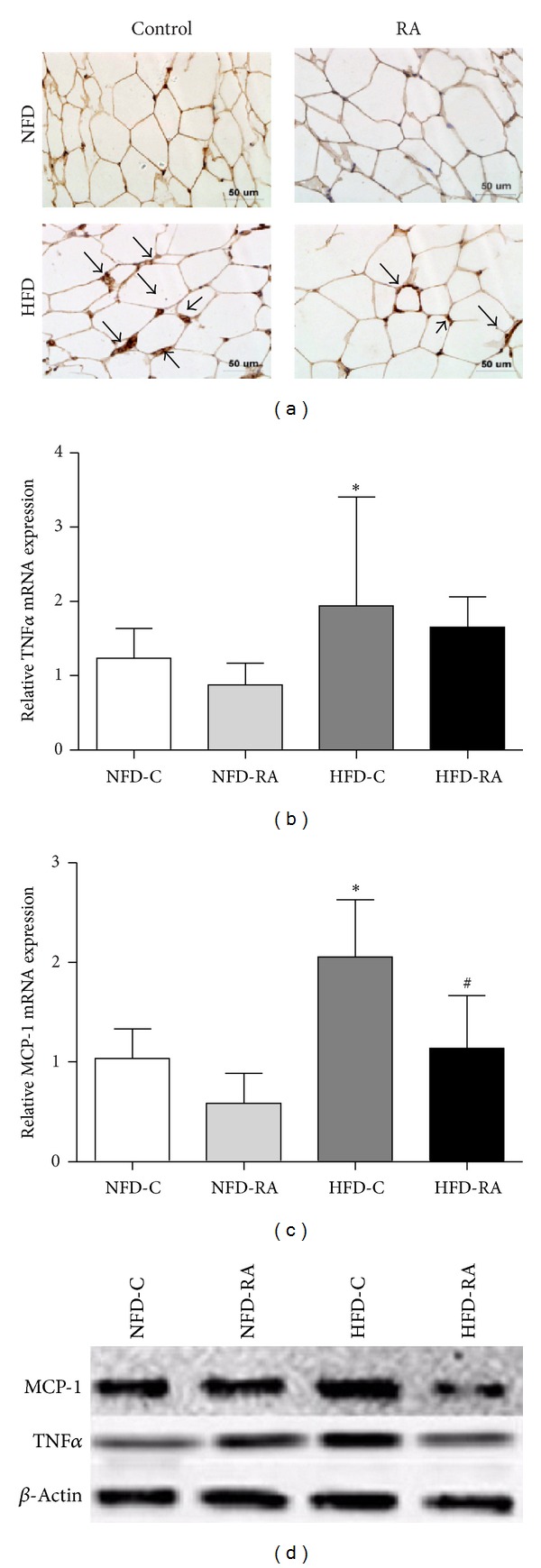
RA treatment improves the inflammatory state in intra-abdominal white adipose tissue. (a) Immunohistochemical staining of epididymal adipose tissue with anti-F4/80 antibodies. qRT-PCR analysis of TNF*α* (b) and MCP-1 (c) in the epididymal fat pad. *P < 0.05 (HFD-C group versus NFD-C group) and ^#^ *P* < 0.05 (HFD-RA group versus HFD-C group). (d) Protein levels of MCP-1 and TNF*α* in the epididymal fat pad.

To elucidate if RA therapy improves the inflammatory state in intra-abdominal white adipose tissue, quantitative real-time PCR, western blot, and immunohistochemistry were performed to evaluate the expression of proinflammatory cytokines and markers of macrophages in epididymal fat pad. RA treatment significantly decreased the mRNA expression and protein levels of MCP-1 in epididymal adipose tissue when compared with the obese rats (Figures 3(c) and 3(d)). Although there was no significant difference in the mRNA expression of TNF*α* between the rats in HFD-C and HFD-RA group, the protein levels of TNF*α* were reduced in the epididymal adipose tissue of the rats that were administered RA orally (Figures 3(b) and 3(d)). In accordance with the expression of proinflammatory cytokines, the expression of F4/80, a marker of mature macrophages, increased in the epididymal adipose tissue of rats fed a high-fat diet and decreased significantly in rats that orally administered RA (Figure 3(a)). These results indicated that RA administration could decrease macrophage infiltration and consequently alleviate the inflammatory state of intra-abdominal white adipose tissue, which is associated with obesity induced by a high-fat diet.

### 3.4. RA Upregulates the Expression of PPAR*γ* in Intra-Abdominal White Adipose Tissue

**Figure 4:**
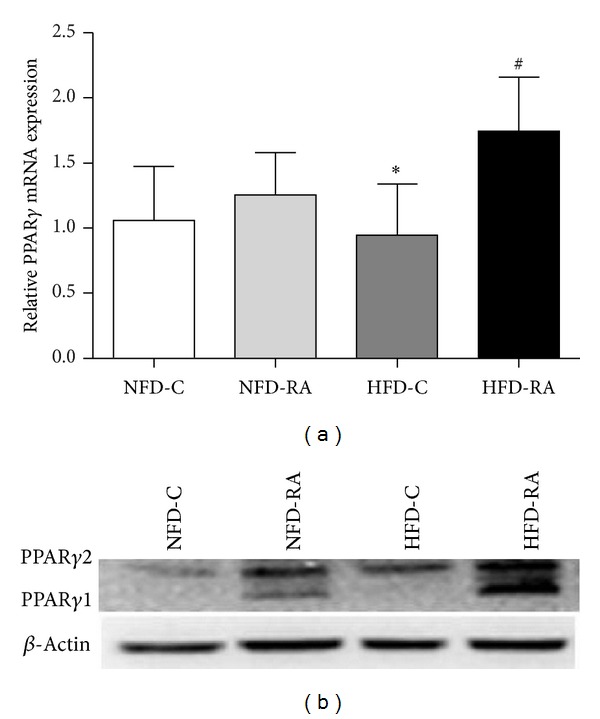
RA upregulates the expression of PPAR*γ* in the intra-abdominal white adipose tissue. (a) mRNA levels of PPAR*γ* from epididymal fat pad were measured by qRT-PCR. *P < 0.05 (HFD-C group versus NFD-C group) and ^#^ *P* < 0.05 (HFD-RA group versus HFD-C group). (b) Protein levels of PPAR*γ* in epididymal fat pad.

To determine whether PPAR*γ* is related to the improved action of RA on dysregulated lipid metabolism and the hyperinflammatory state of intra-abdominal white adipose tissue in obese rats, we detected the PPAR*γ* protein content in epididymal adipose tissue by quantitative real-time PCR and western blotting. As compared with obese rats, PPAR*γ* mRNA and protein levels were significantly increased in the epididymal adipose tissue from RA treated rats (Figures 4(a) and 4(b)).

### 3.5. mTORC1 Signaling Pathway Plays an Important Roles in Upregulating PPAR*γ* Expression by RA

**Figure 5:**
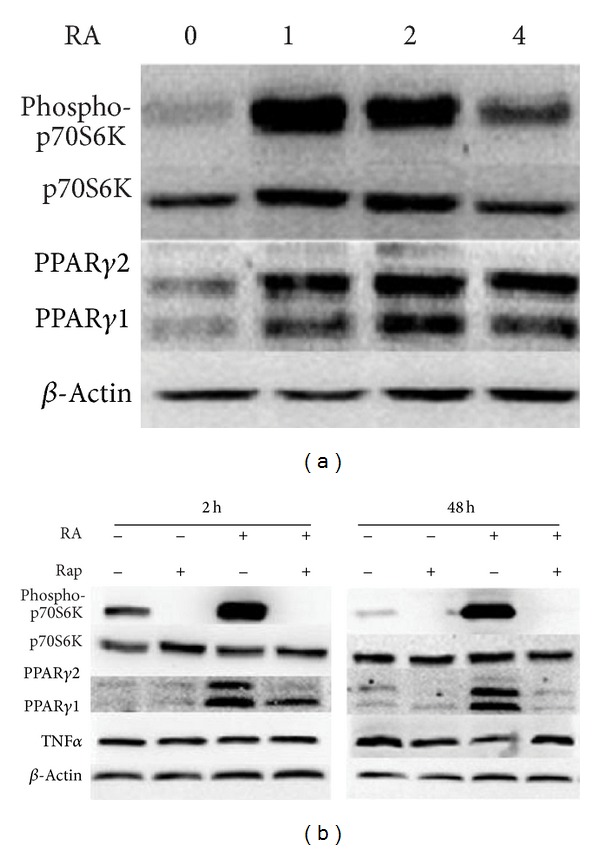
The mTORC1 signaling pathway plays an important role in the upregulation of PPAR*γ* expression by RA. (a) Differentiated 3T3-L1 cells were cultured with 0, 1, 2, and 4 mg/mL RA for 48 hours. Cell extracts were then subjected to western blot analysis with antibodies specific for total and phosphorylated p70S6K (Thr389), PPAR*γ*, and *β*-actin. (b) Differentiated 3T3-L1 cells were cultured with rapamycin (0 or 30 nM) and RA (0 or 2 mg/mL) for 2 or 48 hours. Cell extracts were then subjected to western blot analysis with antibodies specific for total and phosphorylated p70S6K (Thr389), PPAR*γ*, TNF*α*, and *β*-actin.

To explore whether upregulated expression of PPAR*γ* by RA was mediated by mTORC1 activity regulation, we cultured differentiated 3T3-L1 adipocytes with different concentrations of RA and the expression of PPAR*γ* and phospho-P70S6K in cell lysate was examined by western blot. As shown in Figure 5(a), the expression of PPAR*γ* was significantly upregulated in differentiated adipocyte cocultured with RA. Also, consistent with the expression of PPAR*γ*, the phosphorylation of p70s6K at Thr389 (Figure 5(a)), which indicated activity of mTORC1, was significantly upregulated in differentiated adipocytes cocultured with RA. To test if the RA-mediated increases in the expression of PPAR*γ* were due to the upregulated activity of mTORC1, we cocultured differentiated 3T3-L1 adipocytes with 2 mg/mL RA and 30 *μ*M rapamycin, an inhibitor of mTORC1. As we expected, after being stimulated for 2 or 48 hours, RA could not upregulate the expression of PPAR*γ* in the presence of rapamycin (Figure 5(b)). Additionally, TNF*α* was suppressed after 48 hours of RA administration, and the suppressed effect of TNF*α* by RA was eliminated in the presence of rapamycin (Figure 5).

## 4. Discussion

Radix astragali (RA), a herb known as Huangqi in China, is frequently used in traditional Chinese medicine. Extracts of RA have been reported to possess cardioprotective [20], antidiabetic [18], anti-inflammatory [21], and immune enhancing properties [22]. Recently, Lam KS reported that the effective fraction isolated from RA alleviates not only glucose intolerance and insulin resistance, but also hypertriglyceridemia with no changes in serum FFAs in db/db diabetic mice [18]. In our current study, we showed that RA treatment resulted in a significant decrease not only in levels of fasting serum triglycerides but also fasting plasma FFA concentrations with no changes in body weight. In addition, the obese rats treated with RA were more capable of handling a lipid challenge.

An elevated fasting plasma FFA concentration, which indicates to a large extent the increased rate of FFA flux from adipose tissue, has been reported in individuals with obesity, insulin resistance, and type 2 diabetes [23, 24]. Prospective epidemiologic studies have suggested that increased plasma FFA concentration is an independent predictor of progression of insulin resistance [25] and type 2 diabetes [23] and is associated with an increased risk of cardiovascular disease and higher carotid intima-media thickness [26]. Although the casual association between hypertriglyceridemia and cardiovascular diseases remains obscure, hypertriglyceridemia typically occurs in conjunction with low HDL concentrations and high levels of small dense LDL particles, atherogenic conditions associated with increased risk of cardiovascular disease. Thus, the positive effects of RA on the dysfunction of adipose tissue, which resulted in decreased levels of fasting serum triglycerides and FFAs, may indicate that consuming RA may reduce the risk of developing certain conditions such as insulin resistance, cardiovascular disease, and type 2 diabetes.

It is well established that obesity is associated with increased infiltration of monocytes into white adipose tissue, which differentiate into the classical activated M1 macrophages [27]. Monocyte chemoattractant protein-1 (MCP-1), which has higher levels of expression in the adipose tissue of obese animals and humans [28], is implicated in this process [27]. Partially resulting from the increased infiltration of macrophages, the expression of TNF*α* is also elevated in adipose tissue in obesity. Because the expression of MCP-1 and TNF*α* is positively correlated to cell size of adipocyte, a critical determinant of adipose tissue function, there is negative correlation between the degree of inflammation and adipose tissue function [29]. Similarly, we reported a reduction in both average adipocyte size and local inflammation in RA treated rats. This suggested that RA administration may have a positive effect on the functions of both global adipose tissue and individual adipocytes.

In our current study, RA administration resulted in improvements in circular parameters related to triglyceride metabolism. In addition to the metabolic improvements, RA had a positive influence on the inflammatory profile of intra-abdominal white adipose tissue. The underlying mechanisms for these improvements are complex. Activation of PPAR*γ*, which results in stimulating adipogenesis and increasing the number of small, younger adipocytes, an increase in the capacity of adipocytes to store lipids and a reduction in inflammation, is likely to be the potential mechanism. Shen et al. reported that RA upregulated PPAR*γ* activity* in vitro* [19]. Consistently, our study showed that the mRNA and protein levels of PPAR*γ* were significantly upregulated in the epididymal adipose tissue of RA treated obese rats. Increased transcription of PPAR*γ* may be one of the reasons why RA could increase protein levels of PPAR*γ*. Additionally, decreased degradation of PPAR*γ* was predicted to be another explanation because RA could increase the protein level of PPAR*γ* in the epididymal adipose tissue of the rats in NFD-C group with no significant increase in PPAR*γ* mRNA levels (Figure 4). As a consequence of increased expression of PPAR*γ*, there were more small adipocytes in the epididymal adipose tissue of RA treated rats than rats in the HFD control group (Figure 1).

Recent studies indicate that mTORC1 activation can drive PPAR*γ* expression and adipogenesis. One study with TSC2-deficient MEF preadipocytes, which result in highly activated mTORC1, provides critical support to the link between mTORC1 activity and PPAR*γ* expression [16]. What is more, diminished mTORC1 activity by Map4k4-depletion, a Ste20 serine/threonine protein kinase that functions as a negative regulator of mTORC1, is accompanied with a decrease in PPAR*γ* protein levels [30]. Based on this evidence, we hypothesized that the upregulated expression of PPAR*γ* by RA was mediated by mTORC1 activity regulation. As expected, in addition to increased PPAR*γ* protein levels, enhanced phosphorylation of p70S6K, a well-known downstream target of the mTORC1 signaling pathway, was observed in differentiated 3T3-L1 cells treated with RA. The link between upregulated mTORC1 activity and RA induced PPAR*γ* expression was confirmed by rapamycin administration.

Our data showed that there was no change in TNF*α* protein levels after 2 hours of RA treatment, but after 48 hours of RA administration the expression of TNF*α* significantly decreased in differentiated 3T3-L1 adipocytes. In addition, RA could not suppress the expression of TNF*α* in the presence of rapamycin. Rapamycin is generally considered to be an inhibitor of mTORC1, but prolonged treatment of cells with rapamycin can also physically disrupt the mTORC2 signaling pathway [31, 32]. Activated mTORC1 [33], as well as mTORC2 [34], is related to the upregulated expression of TNF*α*. Because of the bidirectional effect of rapamycin on mTOC1 and mTORC2, we could not definitively conclude that the activation of the mTORC1 signaling pathway was the unique mechanism involved in the suppressed effect of RA on TNF*α* expression. Additionally, PPAR*γ* is also involved in regulating the expression of TNF*α* [14]. Therefore, the definite underlining mechanism of the decreased expression of TNF*α* by RA warrants further investigation.

Here, we come to a conclusion that RA improves dysregulated triglyceride metabolism and attenuates macrophage infiltration in adipose tissue in high-fat diet-induced obese male rats through activating mTORC1-PPAR*γ* signaling pathway. However, there are still some limitations in our paper. Firstly, activated mTORC1 by RA increased the mRNA and protein levels of PPAR*γ*, but it is still unknown whether decreased degradation of PPAR*γ* is also involved in the increased protein level of PPAR*γ* by RA. Secondly, the expression of TNF*α* significantly decreased in epididymal adipose tissue from the obese rats treated with RA and the differentiated 3T3-L1 adipocytes cocultured with RA, but whether activated mTORC1 and PPAR*γ* signaling pathway by RA are involved in regulating expression of proinflammatory factors such as TNF*α* and in macrophage function and whether RA affects macrophage polarization are still unclear and warrant further investigations.

## 5. Conclusion 

In summary, this study shows that RA administration results in low levels of fasting triglycerides and FFAs and decreased infiltration of macrophages in intra-abdominal white adipose tissue in high-fat diet-induced obese rats. Additionally, we demonstrated that mTORC1 activation by RA treatment led to enhanced expression of PPAR*γ* in differentiated 3T3-L1 cells. Taken together, the mTORC1-PPAR*γ* signaling pathway is a potential mechanism through which RA exerts beneficial effects on the disturbances in triglyceride metabolism and chronic inflammation of intra-abdominal white adipose tissue in high-fat diet-induced obese rats.
